# Partial Mitochondrial Genome Sequences of Two Abyssal Sponges (Porifera: Hexactinellida), Bathydorus laniger and Docosaccus maculatus

**DOI:** 10.1128/genomeA.00234-18

**Published:** 2018-04-19

**Authors:** Amanda S. Kahn, Jonathan B. Geller

**Affiliations:** a Moss Landing Marine Laboratories, Moss Landing, California, USA

## Abstract

We announce the nearly complete mitochondrial genome sequences of two hexactinellid sponges, Bathydorus laniger and Docosaccus maculatus. A contiguous region of over 15,000 bp was sequenced from each genome. An uncommon structural element was identified as a series of repetitive elements with sequences matching *cob* in the genome of D. maculatus.

## GENOME ANNOUNCEMENT

We sequenced the nearly complete mitochondrial genomes of two abyssal sponges (phylum Porifera), bringing the total coverage to 13 hexactinellid genomes. Tissue samples from the holotypes of Bathydorus laniger Kahn et al. 2013 and Docosaccus maculatus Kahn et al. 2013 were collected in 2007 from Station M, 200 km west of Point Conception, CA (4,100 m depth [1]). Tissue samples were frozen in liquid nitrogen and stored at −80°C. DNA was extracted under liquid nitrogen according to DNeasy animal and blood tissue extraction kit protocols (Qiagen, USA). Mitochondrial DNA (mtDNA) was sequenced using primer walking with standard PCR, long PCR (2), and restriction digestion and cloning. Sequences were assembled using Geneious version 5.3 (3). Coding regions were identified using BLAST searches (GenBank), followed by a comparison of gene translations in Geneious. tRNA sequences were predicted using tRNAscan-SE (4).

We sequenced 15,704 bp for Bathydorus laniger, 14,709 bp of which (93.7%) represents coding regions; the total A+T content was 71.3%. This included 29 genes, with 12 protein-coding genes (three units of cytochrome oxidase [*cox1, cox2,* and *cox3*], six subunits of NADH dehydrogenase [*nad1, nad2, nad3, nad4, nad4L,* and *nad5*], cytochrome *b* [*cob*], and two subunits of ATP synthase [*atp6* and *atp9*]), small (*rns*) and large (*rnl*) subunit rRNAs, and 15 tRNAs. The largest noncoding space was 262 bp long, with no notable elements.

The coverage of the mitochondrial genome of Docosaccus maculatus was similar, with a single contiguous sequence of 17,143 bp, of which 15,212 bp (88.7%) represents coding regions; the A+T content was 70.9%. There were 32 genes, with the same 13 protein-coding genes as B. laniger plus NADH dehydrogenase subunit 6 (*nad6*), *rns,* and *rnl*, and 17 tRNAs.

In both species, most genes used ATG as a start codon, but the start codon for *atp9* was ATA. The stop codons were either TAA or TAG. In D. maculatus, a +1 frameshift occurred in *nad2* (amino acid position 63). The insertion was preceded by the UGG codon for tryptophan, which was present only there and once in *cox1* of B. laniger; all other tryptophan residues were encoded by the more common UGA codon. Interestingly, the UGG codon has been associated with a +1 frameshift in coding regions of other glass sponges (5–7).

Noncoding mtDNA for D. maculatus was concentrated in a 1,439-bp region between *nad1* and *cob*. It contained two stuttering repeats of *trnY* and the 5ʹ end of a partial *cob* sequence upstream of a functional *cob* gene. The region also included two nonfunctional copies of *trnY* (*gua*) and a third functional gene (predicted using tRNAscan-SE [4]) which differed by only a few base pairs from the other two. Repetitive regions have been found in demosponges as palindromic repeats (8–10) but have not been observed in hexactinellid species, and stuttering repeats of protein-coding genes have not been observed in any other sponge genomes; the feature is so far unique to D. maculatus.

### Accession number(s).

Sequences have been deposited in DDBJ/ENA/GenBank under the accession numbers KJ634155 (Bathydorus laniger) and KJ634156 (Docosaccus maculatus).
